# Decitabine reactivated pathways in platinum resistant ovarian cancer

**DOI:** 10.18632/oncotarget.1961

**Published:** 2014-05-13

**Authors:** Fang Fang, Qingyao Zuo, Jay Pilrose, Yinu Wang, Changyu Shen, Meng Li, Phillip Wulfridge, Daniela Matei, Kenneth P. Nephew

**Affiliations:** ^1^ Medical Sciences, Indiana University School of Medicine, Bloomington, IN, USA; ^2^ Department of Endocrinology, Beijing Jishuitan Hospital, Beijing, P.R. China; ^3^ Department of Biostatistics, Indiana University, Indianapolis, IN, USA; ^4^ Computational Biology & Bioinformatics, Indiana University, Indianapolis, IN, USA; ^5^ Norris Medical Library, University of Southern California, Los Angeles, CA, USA; ^6^ Indiana University Melvin and Bren Simon Cancer Center, Indianapolis, IN, USA; ^7^ VA Roudebush Hospital, Indianapolis, IN, USA; ^8^ Department of Obstetrics and Gynecology; ^9^ Department of Medicine, Indiana University School of Medicine, Indianapolis, IN, USA; ^10^ Department of Cellular and Integrative Physiology, Indiana University School of Medicine, Indianapolis, IN, USA

**Keywords:** platinum resistant ovarian cancer, decitabine, gene expression, chemosensitization, DNA methylation, pathway analysis

## Abstract

Combination therapy with decitabine, a DNMTi and carboplatin resensitized chemoresistant ovarian cancer (OC) to platinum inducing promising clinical activity. We investigated gene-expression profiles in tumor biopsies to identify decitabine-reactivated pathways associated with clinical response. Gene-expression profiling was performed using RNA from paired tumor biopsies before and 8 days after decitabine from 17 patients with platinum resistant OC. Bioinformatic analysis included unsupervised hierarchical-clustering, pathway and GSEA distinguishing profiles of “responders” (progression-free survival, PFS>6months) and “non-responders” (PFS<6months). Functional validation of selected results was performed in OC cells/tumors. Pre-treatment tumors from responders expressed genes associated with enhanced glycosphingolipid biosynthesis, translational misregulation, decreased ABC transporter expression, TGF-β signaling, and numerous metabolic pathways. Analysis of post-treatment biopsies from responders revealed overexpression of genes associated with reduced Hedgehog pathway signaling, reduced DNA repair/replication, and cancer-associated metabolism. GO and GSEA analyses revealed upregulation of genes associated with glycosaminoglycan binding, cell-matrix adhesion, and cell-substrate adhesion. Computational findings were substantiated by experimental validation of expression of key genes involved in two critical pathways affected by decitabine (TGF-β and Hh). Gene-expression profiling identified specific pathways altered by decitabine and associated with platinum-resensitization and clinical benefit in OC. Our data could influence patient stratification for future studies using epigenetic therapies.

## INTRODUCTION

Development of chemotherapy resistance is the predominant cause of treatment failure and death in high-grade serous ovarian cancer (OC). The majority of women with OC respond well to initial platinum-based therapy [1], however, over 80% eventually develop platinum resistance, which is uniformly fatal [2]. Therapeutic options are limited for patients with platinum resistant OC and new agents including anti-angiogenics, PARP inhibitors, folate targeted therapies and others are currently under clinical investigation [1]. “Personalized medicine” aiming to tailor therapy to the molecular characteristics of individual tumors has not yet materialized in improved outcomes [3]. While several second-line therapeutic approaches have prolonged progression-free survival (PFS), the impact on overall survival remains modest [4].

Platinum resistance in OC is believed to be multifactorial, resulting from transmembrane drug efflux, impairment of DNA mismatch repair, apoptosis, and senescence-promoting pathways, and/or gain of base-excision repair, growth-promoting, and metabolic pathways [5]. Epigenetic anomalies, including aberrant DNA methylation, histone modifications and nucleosome remodeling, are considered hallmarks of all stages of cancer development and have been consistently described in OC [6]. Specifically, altered DNA methylation patterns, such as increased DNA methylation within CG-rich (“CpG islands”) promoter regions (often within tumor suppressor genes), are a well-studied transcriptionally repressive epigenetic modification widely reported in OC [7, 8]. Recently it has been demonstrated that the “deep silencing” epigenetic mark can be reversed using pharmacological approaches, such as by DNA methyltransferase inhibitors (DNMTIs). We [9, 10] and others [11] previously demonstrated in preclinical studies that decitabine, a DNMTI approved for clinical use, resensitizes chemoresistant OC cells to platinum. We further translated these observations into a phase I/II clinical trial which showed that low-dose decitabine, administered for five consecutive days (qd × 5) followed by carboplatin, resulted in promising clinical activity. Specifically we reported that the regimen induced an objective response rate of 35% in platinum-resistant OC patients [12, 13]. Additionally, 9 of 17 treated patients (53%) were free of disease progression at 6 months.

To determine the effects of the DNMTI on gene expression, we analyzed tumor material obtained either by biopsy or by collection of cell pellets from malignant ascites obtained before (baseline) and after 5 days of decitabine treatment. Integrated bioinformatics analyses correlated decitabine-induced alterations in gene expression and DNA methylation with clinical benefit (PFS > 6 versus < 6 months). Specifically, we sought to identify epigenetically regulated signaling pathways and biological processes associated with decitabine-mediated OC resensitization to platinum resulting in clinical benefit.

## RESULTS

### Gene Set Enrichment Analysis (GSEA) identifies “predictive” profiles and specific genes in pre-treatment (“baseline”) samples

We recently reported the results of a phase I/II trial using low-dose decitabine therapy to resensitize platinum-resistant ovarian tumors to carboplatin [12]. Specifically, the regimen consisted of five consecutive-day, one-hour infusions of 10 mg/m^2^ decitabine (days 1 – 5), followed by carboplatin (AUC 5) on day 8 of a 28-day cycle [12, 13]. Out of 17 enrolled patients, nine were free of disease progression at six months. Moreover response correlated with *in vivo* decitabine bioactivity, as measured by hypomethylation of specific genes/loci (“methylomic” alterations induced by decitabine) [14-16].

**Figure 1:**
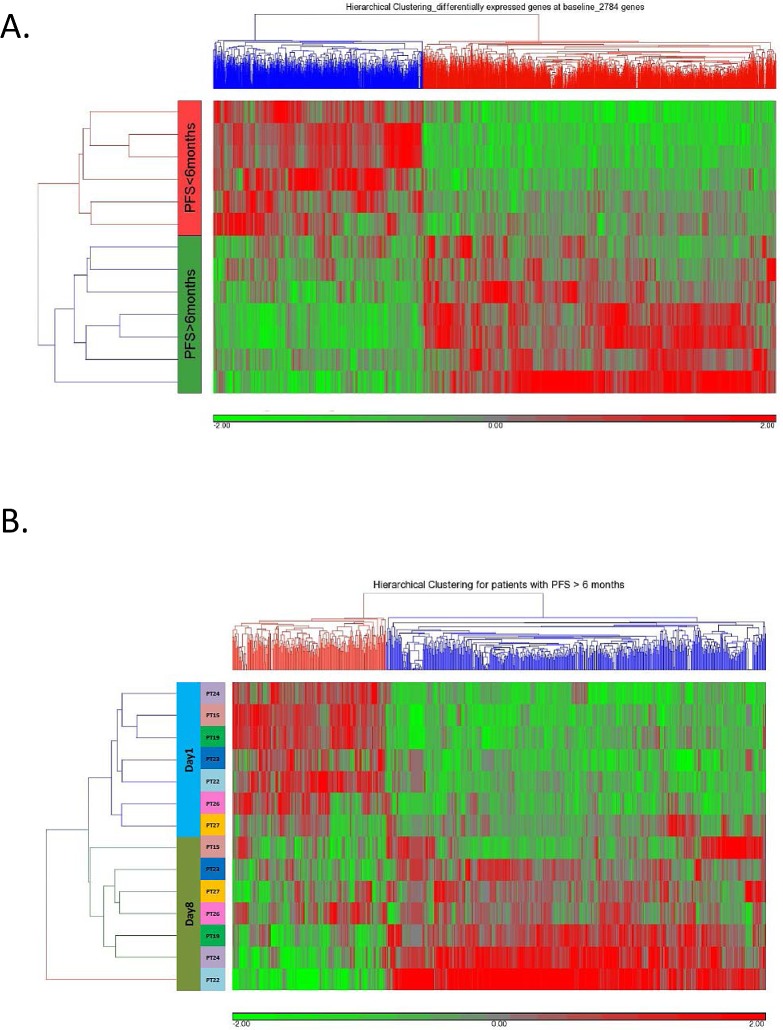
(A) Unsupervised hierarchical clustering of 2784 genes differentially predictive (*P*<0.05) of OC patient response (PFS > 6 months) or lack of response (PFS<6 months) to treatment with one 28-day cycle of 10 mg/m^2^ iv decitabine (qd × 5 days, days 1-5) followed by AUC5 iv carboplatin (qd, day 8). (**B)** Unsupervised clustering of the expression of 744 differentially expressed (528 upregulated, FC > 1.2 and 216 downregulated, FC < −1.2) genes following (day 8) qd × 5 day 10 mg/m^2^ iv decitabine in responsive patients.

We analyzed gene expression profiles in tumor biopsies and cell pellets from malignant ascites pre-decitabine treatment. As shown in Figure 1A, significant difference in pretreatment gene expression patterns was observed between “responders” (PFS > 6 months) and “non-responders” (PFS < 6 months).

Focusing on specific genes identified by GSEA that were enriched in pretreatment tumor biopsies, we noted upregulation of numerous cancer/testes antigen genes (G antigen 12B - *GAGE12B*, X antigen family member 5 - *XAGE5*, synovial sarcoma X breakpoint 10 - *SSX10*, and several melanoma antigen (*MAGE*) family members, encoding tumor-specific cell surface antigens (Supplemental Table S1)) in eventual responders [17].

### Decitabine-induced alterations in gene expression patterns/pathways

To identify specific decitabine-associated alterations of genes/pathways previously associated with OC, we performed bioinformatics analyses to compare changes in gene set expression pre- (day 1) and post- (day 8) decitabine administration in responders and non-responders, respectively. Hierarchical clustering showed differential gene expression in responders (Figure 1B) and non-responders (Supplemental Figure S1). As shown in Table 1, we identified in tumor biopsies from responding patients, decitabine-associated upregulation of antagonists of both TGF-β (chordin - *CHRD*, inhibin β A - *INHBA*,) and Hh (incontinentia pigmenti1 - *IP1*, patched 2 - *PTCH2*) pathways [18, 19]. In addition, downregulation of the TGF-β agonists E1A binding protein 300 (*EP300*), retinoblastoma-like 1 (*RBL1I*), and SMAD specific E3 ubiquitin protein ligase 1 (*SMURF1*) [19, 20], occurring simultaneously with downregulation of gene members of the cell cycle and DNA replication were observed in tumors from responders. These observations suggest that inactivation of the TGF-β pathway by treatment with a DNMTI can be predictive of clinical response.

**Table 1:** KEGG pathways significantly (P<0.05) up- (485 genes, FC>1.2) or down- (187 genes, FC<−1.2) regulated, post-decitabine, in responders

Pathway	Enrichment Score	Enrichment *P*-value	Genes in Pathway (Fold-Change)
TGF-beta signaling pathway	3.13971	0.043295	*EP300* (−1.35), *RBL1* (−1.33), *SMURF1* (−1.31), *CHRD* (1.21), *INHBA* (1.56), *BMP5* (1.34)
Hedgehog signaling pathway	3.85726	0.021126	*HHIP* (1.71), *PTCH2* (1.24), *WNT5B* (1.27)
Cell Cycle	8.96391	0.000128	*CHEK1* (−1.48), *DBF4* (−1.39), *EP300* (−1.35), *MCM5* (−1.53), *MCM6* (−1.49), *PLK1*(−1.60), *RBL1*(−1.33)
Non-homologous end-joining	5.09595	0.006121	*MCM6* (−1.49), *PLK1* (−1.6), *RBL1* (−1.33)
Spliceosome	3.48431	0.0306748	*LSM2* (−1.327), *NHP2L1* (−1.333), *PHF5A* (−1.389), *SNRPF* (−1.568)
Cysteine and methionine metabolism	3.1934	0.0410321	*AHCYL2* (−1.244), *ENOPH1* (−1.325)
Ribosome biogenesis	5.18261	0.005613	*NHP2* (−1.50672), *NHP2L1* (−1.33322), *NOP10* (−1.51841) *POP7* (−1.36675)
DNA replication	3.14207	0.043193	*MCM5* (−1.52675), *MCM6 (−1.49)*
Fructose and mannose metabolism	3.14207	0.043193	*GMD5* (−1.362), *HK1* (−1.354)

In all patients' biopsies, decitabine treatment markedly (P<0.01) altered methylation of CATG1B cancer/testis antigen 1B (*NY-ESO-1*) (Supplemental Figure S2), a target antigen for immunotherapy [21], suggesting a dual role of decitabine as an epigenetic modulator and possible immunosensitizer. Although a vaccine target in OC, limited *NY-ESO-1* expression represents a barrier to vaccine efficacy, and decitabine-mediated upregulation of *NY-ESO-1* has the potential to augment this therapeutic approach [22]. Another well-known gene family found to be overexpressed in non-responders prior to decitabine was tissue inhibitor of matrix metalloproteinases *(TIMP*) class (Supplemental Table S1). Elevated *TIMP1* expression level was also associated with drug resistance in breast cancer [23].

Similar to the GSEA pathway analyses described above, we determined which specific Gene Ontology (GO) terms were enriched in responders and non-responders based on the decitabine-altered gene expression patterns (Supplemental Figures S3 and S4, respectively). In responders, we found enrichment of upregulated GO terms related to glycosaminoglycan binding (in contrast to decitabine downregulation of glycan degradation, Table 1) and other extracellular matrix-protein interactions (purple arrowhead), while enriched downregulated GO terms again included numerous processes related to DNA replication fidelity, cell cycle checkpoints, and mitotic progression (yellow arrowhead) and binding of the oncoprotein nuclear factor kappa B (NF-kappaB) (Supplemental Figure S3).

In non-responders, enrichment of GO terms associated with physiological homeostasis was observed, including endocytosis, transcription and gene expression fidelity (Supplemental Figure S4, gray arrowhead). Downregulated GO terms, by contrast, included various responses related to DNA integrity and its influence on cell cycle progression (thus suggesting attenuated cellular detriment by DNA damage and extracellular matrix (ECM) interactions/cell motility (Supplemental Figure S4, cyan and purple arrowheads, respectively), in similarity to our KEGG-pathway determinations (Supplemental Table S3). Thus, these processes might be expected to be related to normal cell homeostasis (endocytosis, transcription, *etc*.), and those associated with the stemness- and drug resistance-associated epithelial-to-mesenchymal transition, thus further contributing to drug resistance [24].

### Experimental validation of decitabine-altered genes in responders and OC cell lines

To substantiate our computational results, we assessed DNA methylation and expression of distinct key genes from the selected KEGG pathways by using pyrosequencing and quantitative RT-PCR (qRT-PCR). Genes were selected based on previous reports suggesting that these genes are regulated by DNA methylation and are linked to responsiveness to chemotherapy. For instance, hypomethylation corresponding to transcriptional upregulation of the Wnt signaling antagonist *AXIN1* [25, 26] has been reported to contribute to chemoresistance in OC. The embryonic developmentally regulated gene homeobox A11 (*HOXA11*) has been shown to play a direct role in platinum resistance and *HOXA11* methylation correlated with suboptimal tumor debulking and OC poor prognosis [27]. The role of the DNA mismatch repair enzyme mutL homolog 1 (*MLH1*) in platinum response in OC has been widely reported [6, 28, 29]. Targeted validation of methylation and gene expression for these transcripts (Supplemental Figure S5).

**Figure 2:**
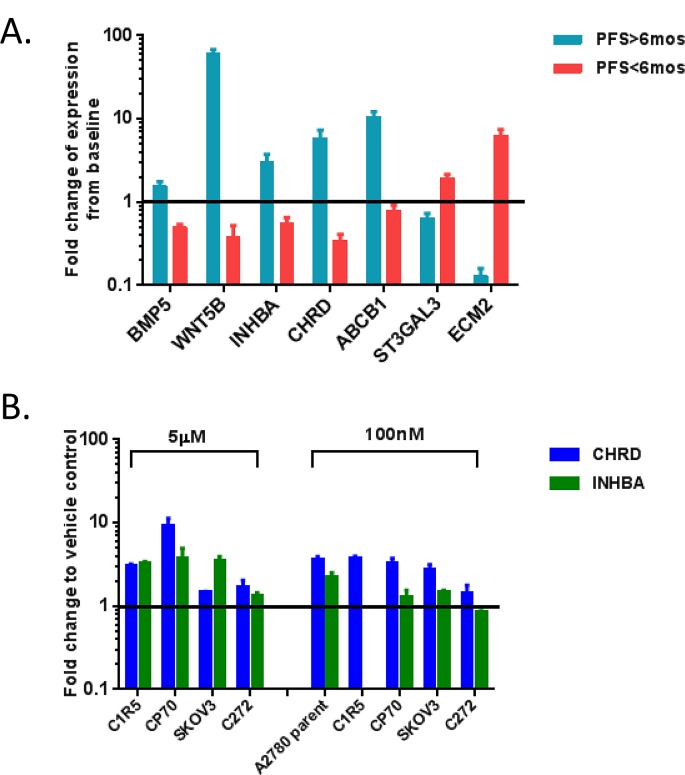
Validation of the microarray for the expression of specific genes in the patient biopsies **(A)** and cell lines (B) was performed by isolation of total RNA, reverse transcription, and quantitative PCR, using the 2^-ΔΔCT^ method of relative quantification. EF1α was used as an internal control. The data was reported as Mean ± SD of three independent experiments in triplicates. Fold changes were calculated by relative expression of pre-decitabine **(A)**, or vehicle control. Responders are shown as PFS>6months, while non-responders are shown as PFS<6months. **(B**). All data shown here are with *P*<0.05, and the black horizontal lines show where the fold change (fc) =1.

We also performed qRT-PCR to validate selected genes from Table 1 and supplemental Table S3 in RNAs from patient biopsies and from OC cell lines treated with decitabine. Genes were selected based on involvement in pathways altered by decitabine induced hypomethylation and known clinical relevance to OC progression. First, we tested bone morphogenetic protein 5 (*BMP5*), wingless-type MMTV integration site family member 5B (*WNT5B*), inhibin beta A (*INHBA*), chordin (*CHRD*), ATP-binding cassette sub-family B member 1 (*ABCB1*), ST3 beta-galactoside alpha-2,3sialytransferase 3 (*ST3GAL3*) and extracellular matrix protein 2 (*ECM2*) in patient biopsies. Consistent with the microarray results, *BMP5*, *WNT5B INHBA*, *CHRD* and *ABCB1* were upregulated in post-treatment samples from responders and downregulated in non-responders. *ST3GAL3* and *ECM2* were upregulated in non-responders (Figure 2A).

Having first taken a bioinformatics approach to identify the TGF-β pathway as responsive to epigenetic interventions, we subsequently used qRT-PCR to measure mRNA expression levels of *CHRD* and *INHBA*, two TGF-β pathway antagonists, in OC cell lines treated with decitabine (A2780, A2780 C1R5, SKOV3, CP70, and C272; Figure 2B). Decitabine treatment upregulated *CHRD* and *INHBA* expression levels in the majority of cell lines examined (5μM decitabine upregulated both genes in C1R5, CP70, SKOV3, and C272, 100nM decitabine upregulated both genes in A2780, CP70, and SKOV3), supporting that the TGF-β pathway is responsive to treatment with DNMTIs.

**Figure 3:**
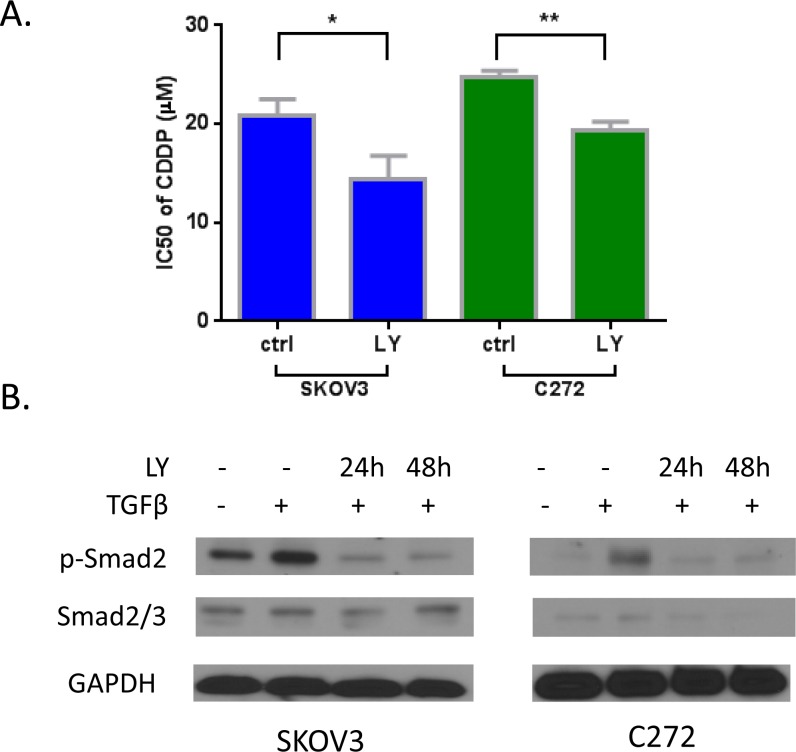
A) Inhibition of TGF-β pathway by inhibitor LY-364947 resensitized cisplatin on SKOV3 and C272 cells. **B)** Activation of p-Smad2 by TGF-β was inhibited by LY-364947 by western blotting.

To demonstrate that the TGF-β pathway is functionally relevant to platinum resistance, we used the TGF-β receptor I antagonist LY-364947. LY-364947 prevented TGF-β induced activation of p-Smad2 (Figure 3A) in these cells. Pre-treatment with the TGF-β receptor inhibitor resensitized SKOV3 and C272 OC cells to cisplatin (Figure 3B), supporting that abnormal activation of the pathway is associated with platinum resistance. These results suggest that decitabine may resensitize ovarian tumors to cisplatin partly by upregulating the expression of antagonists in the TGF-β pathway.

### Integration of decitabine-induced changes in DNA methylation and gene expression

**Figure 4:**
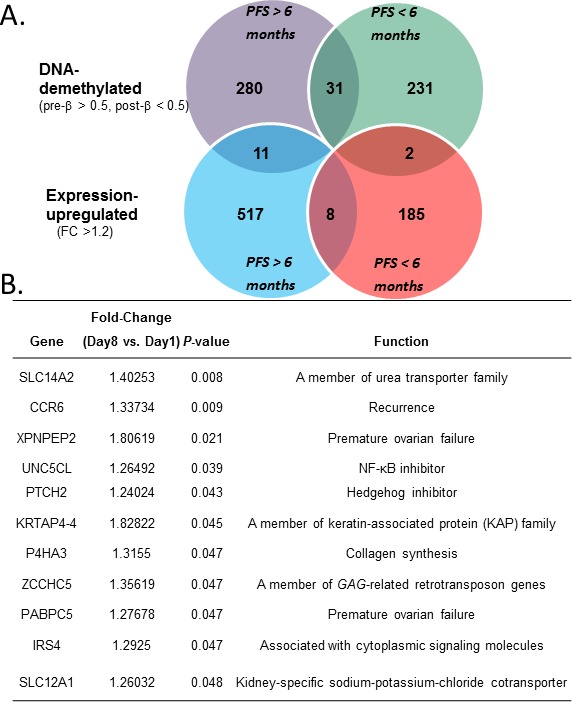
(A) Overlap of genes showing DNA methylation significantly (*P*< 0.05) negatively correlated with gene expression in responders (PFS > 6 months, left circles) and non-responders (PFS < 6 months, right circles). (**B)** Details of the 11 genes found hypomethylated and upregulated in responders.

To integrate “methylome” and “genome” changes, we correlated gene expression and DNA methylation alterations by using the Partek Genome Suite. In responsive patients (PFS>6 months), hypomethylation of 311 genes was observed (Figure 4A), with 11 genes demonstrating concurrent upregulation (Figures 4A, 4B). Of specific interest was the putative tumor suppressor *PTCH2* [30], an antagonist of the Hh pathway, a pathway our previous comprehensive analysis of methylation profiles found to be of particular importance to poor OC survival [31]. Hh pathway activity plays a key growth-promoting role in various malignancies [32]. *PTCH1* and *PTCH2*, coding two homologous Ptch receptors, have been shown to interact with Hh ligands and regulate signaling through the Hh pathway [33]. Our results showing that *PTCH2* can be demethylated and its expression upregulated by decitabine (also see Supplemental Table S1) suggest for the first time a role for *PTCH2* in epigenetic chemoresensitization in OC patients.

Additional genes found to be concurrently DNA-hypomethylated and upregulated (Figure 4B) included those belonging to the solute carrier (SLC) family, encoding transporters involved in drug uptake/efflux and attenuated isoosmolarity (solute carrier family 14 member 2 - *SLC14A2* and solute carrier family 12 member 1 - *SLC 12A1*), chemokine receptor 6 (*CCR6*), unc-5 homolog C-like (*UNC5CLII*; also known as *ZUD*), prolyl 4-hydroxylase, alpha polypeptide III (*P4HA3*), poly(A) binding protein cytoplasmic 5 (*PABPC5*), and insulin receptor substrate 4 (*IRS4*). The potential implications of these observations are discussed below.

While 11 genes were hypomethylated and upregulated in responders (Supplemental Figure S6A), only two such genes were identified in non-responders, IQ motif containing F2 (*IQCF2*) and EF-hand calcium binding domain 3 (*EFCAB3*) (Figures 4A, Supplemental Table S4). Neither of them has been reported to be associated with cancer or drug resistance. As shown in Supplemental Figure S6B, these genes continued to remain upregulated in all non-responsive patients examined, following decitabine treatment (day 8), as compared to pre-treatment (day 1). Collectively, the findings from this small study may represent a potential panel of genes for further investigation as prognostic biomarkers of hypomethylating strategies in OC.

## DISCUSSION

Previously, we thoroughly assessed alterations in global DNA methylation in rapport to clinical outcomes in a phase II clinical trial of decitabine and carboplatin for patients with advanced-stage, recurrent and platinum-resistant OC [12, 13]. In the current work, *in silico* bioinformatics analyses integrate transcriptomic and methylomic data from tumor biopsies obtained before and after treatment. Our results delineate candidate signaling pathways and biological processes associated with clinical outcomes (PFS>6 months vs. <6months) in this trial. To our knowledge, this represents the first study assessing gene expression in human solid tumors exposed to a hypomethylating agent and the first attempt to identify profiles predictive of clinical benefit. Our analyses demonstrate highly divergent patterns of gene expression in responders vs. non-responders, both at baseline (pre-treatment) and following decitabine administration. We also describe alterations in specific pathways/biological processes induced by decitabine in tumor samples in association with clinical response in a small study.

Specifically, we found that pre-treatment (“baseline”) expression of several members of the cancer/testis antigen and TIMP gene families were predictive of response (Supplemental Table S1). TIMP3 expression was previously associated with metastasis and poor survival in OC [34], while re-expression of the embryonically expressed, but subsequently repressed cancer antigens may be associated with an immune response caused by decitabine [22]. This is particularly intriguing as decitabine elicited higher than expected rates of platinum hypersensitivity reactions in this clinical trial [13], consistent with an immune-mediated effect. These observations highlight the possibility that the anti-tumor effect of the combination treatment may result from heightened immune reaction activated by the hypomethylating strategy.

In tumor samples obtained following decitabine therapy, up-regulation of Hh antagonists, in concert with downregulation of gene members involved in nonhomologous end joining, cell cycle, and numerous metabolic pathways (Table 1) was associated with clinical outcome. These findings provide a rationale for future testing of decitabine and Hh antagonists combinations in OC models [35]. The data are also consistent with data from our recent study demonstrating that Hh pathway is a key signaling pathway regulated by DNA methylation in OC and that loss of negative Hh signaling feedback may contribute to disease progression [31].

Likewise, in post-decitabine biopsies of responders, downregulation of TGF-β signaling was observed, a finding we substantiated in cell-based assays. We demonstrated that inhibition of TGF-β signaling by using a receptor kinase inhibitor resensitized OC cells to platinum; suggesting that inactivation of the pathway by decitabine in our trial could have contributed to the observed clinical response to carboplatin. The findings are in agreement with our and others' results implicating TGF-β pathway in ovarian tumor progression [36, 37]. We have also previously reported that abrogated responses to TGF-β in OC cells are associated with epigenetically mediated gene silencing [38].

Interestingly, we observed the involvement of a less reported, OC-associated epigenetic phenomenon, *i.e.*, glycan metabolism and protein/lipid glycosylation patterns [39], noting increased biosynthesis/decreased degradation associated with baseline and post-treatment response (Table 1), and decreased synthesis linked to nonresponse (Supplemental Table S3). These results concur with other reports that glycosylation patterns can significantly affect tumor-suppressive vs. tumor-promoting effects of glycoproteins [40], and that N- and O-glycans may facilitate tumor migration and metastasis [40].

The integrated analysis seen in Figure 4 revealed a potential role for several decitabine-derepressed genes (hypomethylated and upregulated) such as *SLC14A2* and *SLC 12A1* encoding transporters involved in drug uptake/efflux and attenuated isoosmolarity [41], *CCR6*, which has been correlated with favorable prognosis in lung cancer patients [42], and *UNC5CLII*, an inhibitor of NF-kappaB activation that sensitized 293 cells to apoptosis [43]. In addition, methylation-dependent silencing of *P4HA3* in B-cell lymphoma cell lines was previously shown to be reversible by DNMTI treatment [44], and *PABPC5* has been associated with premature ovarian failure [45] but a role for either gene in OC has not been previously reported. Finally, although enhanced cell proliferation and Wnt/β-catenin signaling by *IRS1/2* was reported [46], *IRS4-*mediated suppression of *IRS1* and *IRS2* has been shown [47, 48], suggesting that decitabine-mediated reactivation of *IRS4* could play an inhibitory role in OC cell proliferation.

The current results concur with our previous report describing genomic changes associated with acquired platinum resistance in an OC cell line rendered resistant by exposure to incremental increases in cisplatin, over five cell generations [10]. In that model we also observed that numerous GO terms encompassing mitotic pathways, DNA repair, oxidative phosphorylation-related processes, and macromolecule biosynthesis being upregulated in platinum-resistant OC cells [10]. Analogously, that system revealed downregulated apoptotic processes and protein kinase inhibition in the resistant cells similar to the pathways enriched in non-responsive tumors[10]. Taken together, the mechanistic results linked to this small phase I/II study are consistent with long-held hypotheses regarding the origin of OC platinum resistance [5, 25, 49], including inhibition of drug efflux, alteration of pro-survival (*i.e.*, anti-apoptosis) pathways, upregulation of Hh antagonists and downregulation of DNA repair/cell cycle progression signaling.

In summary, this study provides strong clinical and biological evidence supporting further investigation of hypomethylating strategies in platinum-resistant OC. We identified numerous pathways linked to chemotherapy response whose baseline activity levels also associate with clinical outcome. These data suggest that not one pathway, but rather complex networks involved in drug sensitivity/resistance mechanisms require “epigenetic reprogramming” to successfully resensitize insensitive tumors to platinum. We show that this can be achieved *in vivo* by using decitabine, a global DNA hypomethylating agent, in combination with chemotherapy. Our results, although based on a small cohort of patients, provide convincing rationale for future testing of combinations regimens including decitabine with other targeted therapeutics (e.g. immune stimulators or TGF-β and Hh inhibitors).

## METHODS AND METHODS

### Patient enrollment, dosing schedule, and collection of patient tumor biopsies

As described in our previous reports [12, 13], eligibility for patient enrollment in the phase II trial included a diagnosis of OC or primary peritoneal carcinomatosis (PPC) and disease progression or recurrance within six months after platinum-based chemotherapy. 28-day treatment cycles consisted of 10 mg/m^2^ intravenous (iv) decitabine (Eisai, Tokyo, Japan), daily (qd) for five consecutive days, followed by iv carboplatin (Bristol Meyers Squibb, Princeton, NJ) administered on day 8 at an area under the curve (AUC) of 5 [12, 13]. Tumor tissue or malignant ascites were obtained through core biopsies or paracentesis under radiographic guidance on days 1 (pre-decitabine) and 8 (post-decitabine, pre-carboplatin) during cycle 1, from enrolled patients, as previously described [12]. Objective response was the primary objective and was measured by RECIST1.0 criteria, while duration of PFS was a secondary endpoint [12].

### Gene expression profiling (pre- and post-decitabine)

Total RNA was extracted from 25 mg tumor tissue using TRIzol reagent (Invitrogen), according to the manufacturer's procedure. cRNA was generated, labeled and hybridized to the Affymetrix (Santa Clara, CA) GeneChip® Human GENE 1.0 ST arrays by the Center for Medical Genomics at the Indiana University School of Medicine (Indianapolis, IN, http://cmg.iupui.edu/), as we have described previously [10]. The hybridized GeneChip® Human GENE 1.0 ST arrays were scanned using a Affymetrix GeneChip Scanner 3000 and analyzed using the Affymetrix Microarray Analysis Suite (MAS) version 5.0. The average density of hybridization signals from independent slides (4 slides per biopsy) was used for data analysis and genes with signal density less than 300 pixels were omitted from the data analysis. Genome-wide DNA methylation analysis using the Infinium HumanMethylation27 BeadChips (Illumina, San Diego, CA) was performed as previously published [12, 13]. The gene expression analysis results are available for download at Gene Expression Omnibus data repository at the National Center for Biotechnology Information (NCBI) under the accession number GSE55410.

### Pathway/biological process data analysis

Partek Genomic Suite (PGS; St. Louis, MO) was used for unsupervised hierarchical clustering of total gene expression and all pathway and gene set analyses, linked to the databases KEGG (Kyoto Encyclopedia of Genes and Genomes, www.genome.jp/kegg/) and GSEA (Gene Set Enrichment Analysis, www.broad.mit.edu/GSEA) [16]. In addition, PGS was used to determine DNA methylation and gene expression statistical correlations.

### Experimental validation of gene expression and methylation

Validation of the microarray results in tumor samples and cell lines was performed by isolation of total RNA (described above), reverse transcription by MMLV reverse transcriptase (Promega, Madison, WI), and quantitative PCR, using the 2^−ΔΔCT^ method of relative quantification, as we have previously described [9, 10] (expression normalization to the housekeeping gene EF1α). Primer sequences are provided in Supplemental Table S2. Pyrosequencing was used to validate gene methylation (EpigenDX, Hopkinton, MA).

### Chemicals and reagents

5-aza-2'-deoxycytidine (decitabine), and TGF-β type I receptor kinase inhibitor LY-364947 (Sigma-Aldrich Co. LLC., St. Louis, MO); glyceraldehyde-3-phosphate dehydrogenase (GAPDH) antibody, and cisplatin (EMD Millipore, Seattle, WA); phospho-Smad2 antibody and Smad2/3 antibody (Cell Signaling Technology, Danvers, MA); recombinant human TGF-β2 (Gibco®, Grand Island, NY) were used.

### Cell culture

OC cell lines included cisplatin-resistant CP70, SKOV3, and A2780 (parent and its cisplatin-resistant subline C1R5) were cultured as previously described [10, 50]. The immortalized cell line C272/hTert/E7 was cultured in growth medium containing 1:1 MCDB 105 (Sigma-Aldrich Co. LLC., St. Louis, MO) and M199 (Cellgro) supplemented with 10% fetal bovine serum (Cellgro) and 1% antibiotics, as described previously [36].

### Drug treatments

To validate the re-expressed genes in cell lines by epigenetic agent, *in vitro* drug treatment and qRT-PCR were performed. Briefly, 48 hours after 10^5^ cells of each OC cell line seeded in the 10cm-dish, different doses of decitabine (100nM for 72 hours or 5μM for 48 hours) was added to the culture. Following drug treatments, RNA was isolated for qPCR validation, which was described previously [10].

### Cell proliferation and immunoblotting assays

MTT assay was performed to evaluate platinum resensitization by TGF-β pathway inhibitor. Detailed experiment procedures were described previously [9, 10]. Briefly, SKOV3 and C272cells were pre-treated for 24-48 hours with 10μM LY-364947 before 3 hour incubation with cisplatin. Cells were lysed in ice-cold Radio-Immunoprecipitation Assay (RIPA) buffer containing protease and phosphatase inhibitor cocktail, EDTA-free (Thermo Scientific, Rockford, IL USA). After sonication and centrifugation, equal amounts of proteins were separated by SDS-PAGE. After electroblotting, the PVDF membranes were incubated with primary and HRP–conjugated secondary antibodies. Immunoreactive proteins were detected by enhanced chemiluminescence solution (Thermo Scientific). Images were captured by a luminescent image analyzer with a CCD camera (LAS 3000, Fuji Film) and quantified by densitometric analysis using Gel-Pro Analyzer 3.1 software.

All the results of *in vitro* studies are reported as means ± SD of at least three independent experiments.

## SUPPLEMENTARY FIGURES, TABLES AND INFORMATION




