# Supplementary material for: Decitabine reactivated pathways in platinum resistant ovarian cancer
Source: Oncotarget. 2014 May 13;5(11):3579–89. doi: 10.18632/oncotarget.1961 (PMC4116504; doi:10.18632/oncotarget.1961)
Supplement: Supplementary file 2 [file oncotarget-05-3579-s002.pdf]

Decitabine reactivated pathways in platinum resistant ovarian cancer

Supplementary Material

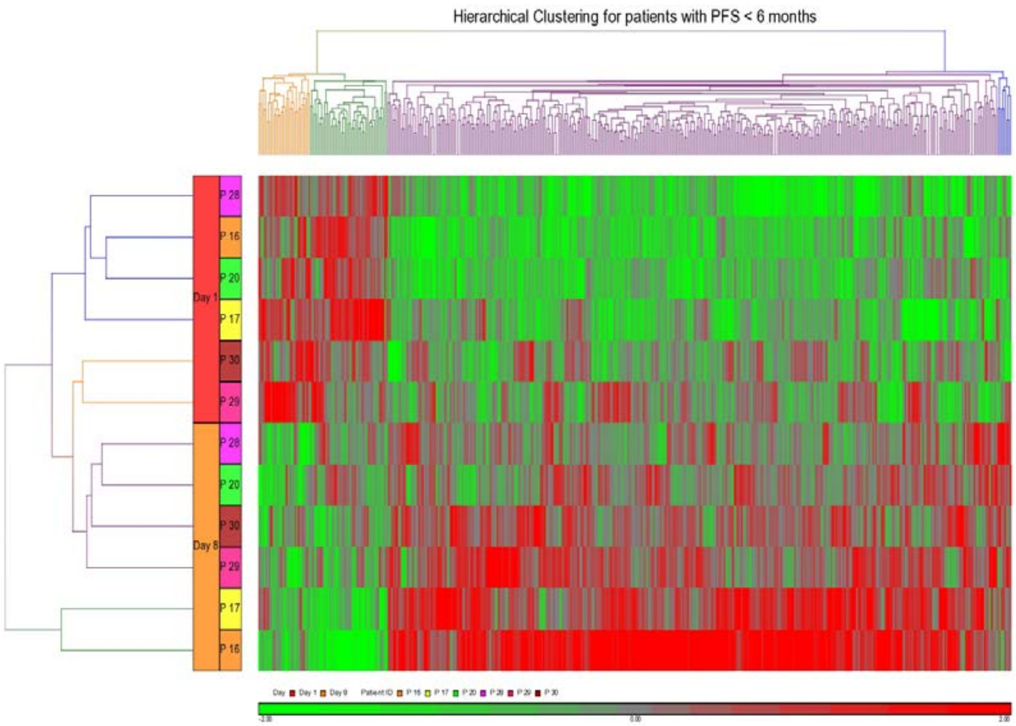

Supplemental Figure S1: Unsupervised clustering of differential gene expression as in (Figure 1A) of day 8 vs. day 1, in non-responsive patients (PFS < 6 months) (69 upregulated genes, 26 downregulated genes).

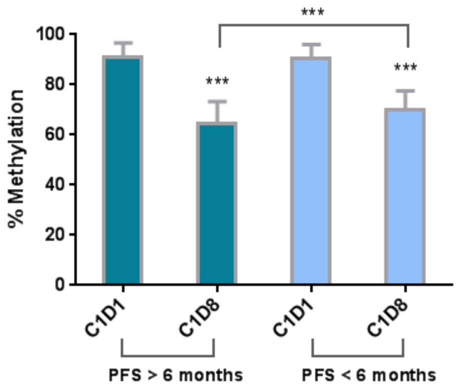

Supplemental Figure S2: Decitabine induces demethyaltion of NY-ESO-1 in OC patients' biopsies. Cycle 1 Day 1 (C1D1) represents baseline level; while C1D8 represents day 8 of the first cycle (decitabine treatment was from days 1-5). Data was reported from 7 responders (shown as PFS > 6 months) and 4 non-responders (shown as PFS < 6 months), and 15 CpG sites were analyzed (***: $P < 0.001$).

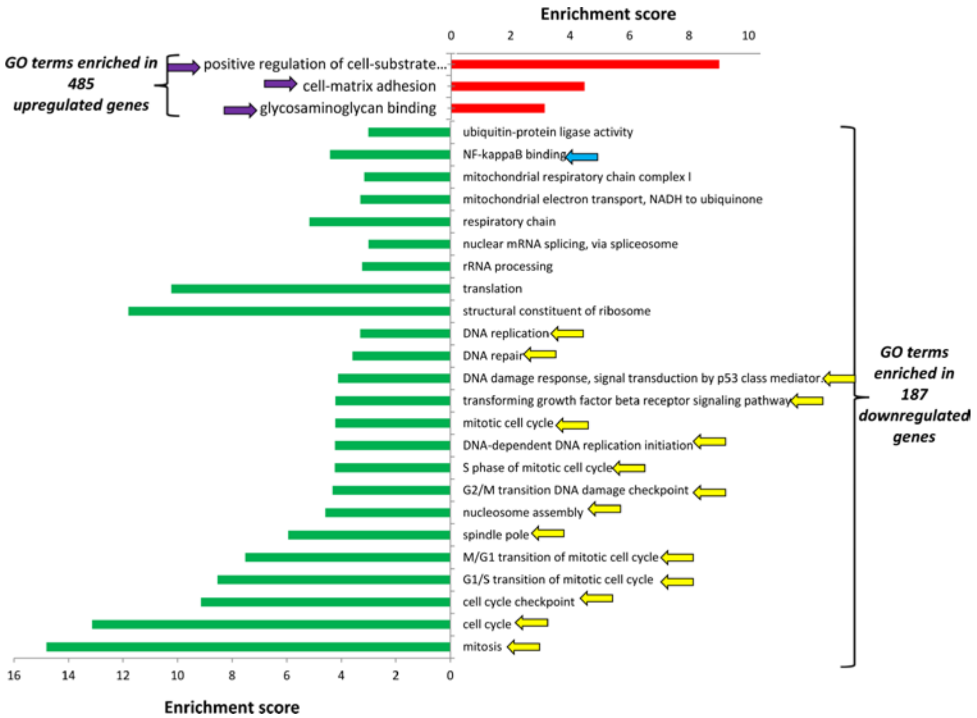

Supplemental Figure S3: Post-decitabine up (of 485 genes, red bars) or down (of 187 genes, green bars) -regulated Gene Ontology (GO) terms in responders. All three upregulated GO terms were related to extracellular matrix interactions (purple arrowhead), while downregulated GO terms included phenomena associated with mRNA processing/ribosomal synthesis/translation (green arrowhead), mitochondrial processes (brown arrowhead) and DNA damage/cell cycle progression (yellow arrowhead).

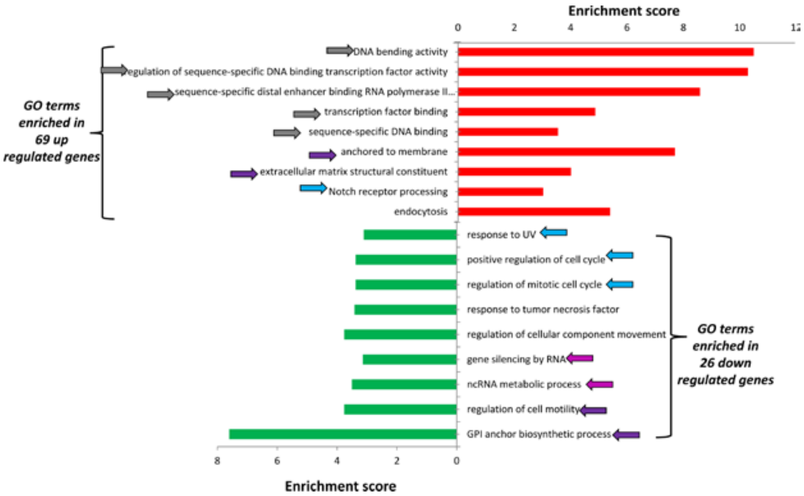

Supplemental Figure S4: Post-decitabine up (of 69 genes, red bars) or down (of 26 genes, green bars) -regulated GO terms in non-responsive patients (PFS < 6 months). Noteworthy upregulated cellular processes included transcriptional homeostasis/functional gene expression (gray arrowhead) and extracellular protein/cellular matrix interactions (purple arrowhead), while downregulated processes included those also associated with extracellular protein/matrix/motility interactions (blue), in addition to various noncoding-RNA-related processes (magenta), and DNA replication/repair cell cycle effects (yellow, also see SF1).

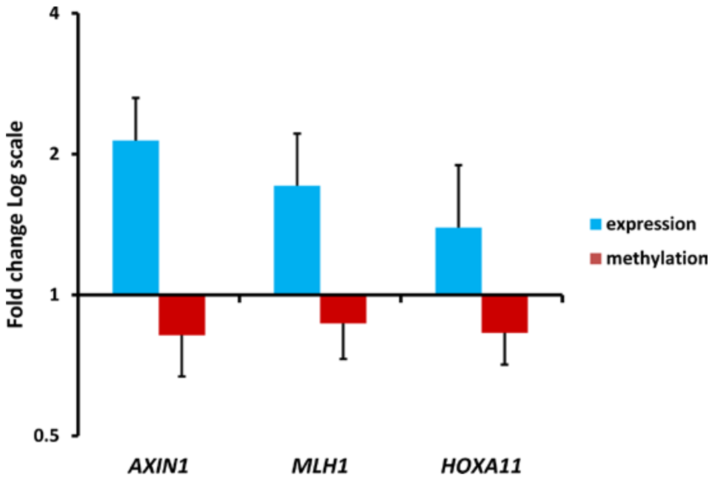

Supplemental Figure S5: Hypomethylation and upregulation of *AXIN1*, *MLH1*, *HOXA11* in A2780 ovarian cancer cells. *P*<0.05 for all data shown.

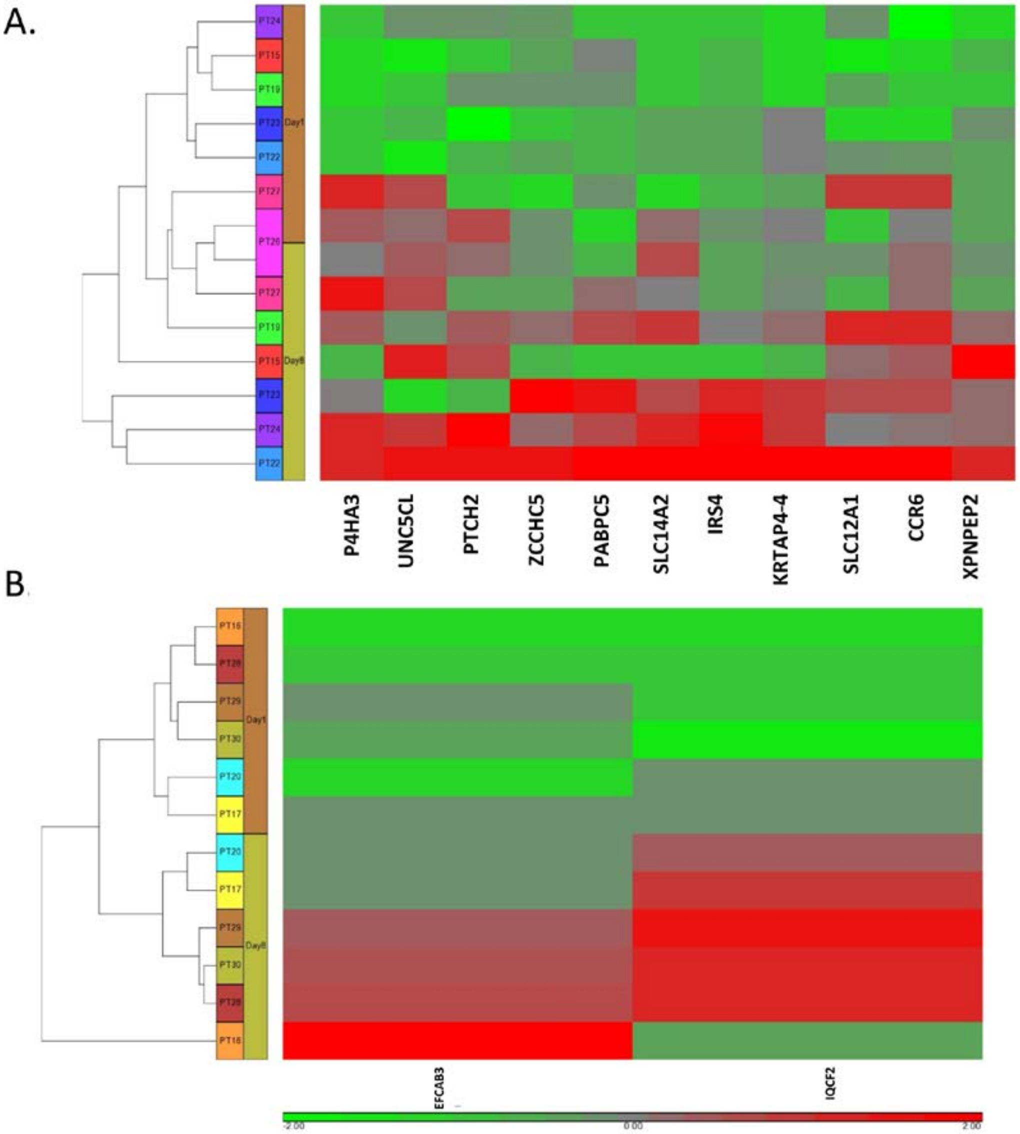

Supplemental Figure S6: Heatmap of unsupervised cluster analysis showing changes in expression of genes hypomethylated and upregulated on pre- (Day 1) and post- (Day 8) decitabine in patients PFS >6 months **(A)**, or patients with PFS < 6 months **(B)**.

Supplemental Table ST2: Primers used for qRT-PCR.

Genes	Forward Primers	Reverse Primers
BMP5	GCTGCTGGGTTCTAGTGGG	TTCGTGGTTCCGTAGTCTTCTA
WNT5B	GCTTCTGACAGACGCCAACT	CACCGATGATAAACATCTCGGG
CHRD	CTAACCCAGGTTCCCTTGAGG	GGCAGCAATGTGTCCACTGA
INHBA	CAACAGGACCAGGACCAAAGT	GAGAGCAACAGTTCACTCCTC
ABCB1	TTGCTGCTTACATTCAGGTTTCA	AGCCTATCTCCTGTCGCATTA
ST3GAL3	GCCTGCTGAATTAGCCACCAA	GCCCACTTGCGAAAGGAGT
ECM2	AACAAAACAAAACTGCAATCTTCA	ATGACACCAAGCAAAGCCTAC
HOXA11	ACACTGAGGACAAGGCCG	GAAGAAGAACTCCCGTTCCA
MLH1	TTAATGAGCAGGGACATGAGG	AACTTGGTTTGATGCTGTGC
AXIN1	CCGGAAGACTCCCTCAGAA	CCGGCATTGACATAATAGGG
EF1α	GCCCCAGGACACAGAGACTTTATC	CAACACCAGCAGCAACAATCAG

Supplemental Table ST3: KEGG pathways from genes significantly (P<0.05) up- (69 genes, FC>1.2) or down- (26 genes, FC<-1.2) regulated, post-decitabine, in non-responders.

Pathway	Enrichment Score	Enrichment *P*-value	Genes in Pathway (Fold-Change)
Focal adhesion	5.08504	0.006189	*COL4A6* (1.21), *COL11A2* (1.35), *PAK3* (1.30)
Protein digestion and absorption	4.55132	0.010553	*COL4A6* (1.21), *COL11A2* (1.35)
ECM-receptor interactions	4.48225	0.011308	*COL4A6* (1.21), *COL11A2* (1.35)
Other types of O-glycan biosynthesis	6.2702	0.001891	*CHST10* (-1.37), *ST3GAL3* (-1.25)
Glycosaminoglycan biosynthesis - keratan sulfate	3.76784	0.023101	*ST3GAL3* (-1.25)
Glycosylphosphatidylinositol (GPI)-anchor biosynthesis	3.2888	0.037298	*PIGO* (-1.31)
Glycosphingolipid biosynthesis- lacto and neolacto series	3.2888	0.037298	*ST3GAL3* (-1.25)

Supplemental Table ST4. Details of the two genes hypomethylated and upregulated in non-responders.

Gene	Fold-Change (Day8 vs. Day1)	*P*-value	Function
IQCF2	1.17503	0.001	unknown
EFCAB3	1.18127	0.020	Calcium-binding protein
